# The gender-specific associations between perceived parenting and sexual-related behaviors among secondary vocational school students in China: a cross-sectional study

**DOI:** 10.3389/fpsyg.2025.1644453

**Published:** 2025-12-05

**Authors:** Simin Xu, Xiayun Zuo, Yan Cheng, Yan Jin, Yuhang Fang, Yujia Zheng, Chunyan Yu, Qiguo Lian, Chaohua Lou, Lihe Li, Ping Hong, Xiaowen Tu

**Affiliations:** 1 NHC Key Lab of Reproduction Regulation, Shanghai Institute for Biomedical and Pharmaceutical Technologies, Shanghai, China; 2 Shaanxi Xin Hang Public Health Research Center, Shaanxi, China; 3 China Family Planning Association, Beijing, China

**Keywords:** parenting, adolescents, sexual behavior, students, psychological control

## Abstract

**Background:**

Although parenting practices are closely linked to adolescent sexual-related behaviors, few studies have been conducted to explore the links between them in Chinese culture, which is characterized by unique familial expectations and conservative values regarding sexuality. Compared to their academic high school counterparts, secondary vocational school students exhibit elevated sexual activity and risk. The study investigates the gender-specific associations between paternal and maternal parenting and sexual-related behaviors among this group.

**Methods:**

A self-report questionnaire was employed to collect data from a cross-sectional study conducted among 3,237 secondary vocational school students from April to June in 2021 in Shanghai Municipality and Shaanxi Province in China. Perceived parenting was assessed using validated scales measuring connectedness, behavioral monitoring, and psychological control. A two-level multivariable logistic regression model was used to examine the associations between perceived parenting and sexual-related behaviors using both variable-centered and person-centered modeling approaches. The latter is based on latent profile analysis that identifies distinct groups of individuals with similar patterns of parenting behaviors.

**Results:**

Variable-centered analysis showed that paternal connectedness was negatively associated with romantic relationships (*aOR* = 0.82, 95%*CI* = 0.71~0.94) and intimate behaviors (*aOR* = 0.83, 95%*CI* = 0.71~0.97) for girls. For boys, paternal and maternal behavioral monitoring were negatively associated with intimate behaviors (paternal: *aOR* = 0.86, 95%*CI* = 0.75~0.98; maternal: *aOR* = 0.83, 95%*CI* = 0.73~0.95) and sexual intercourse (paternal: *aOR* = 0.69, 95%*CI* = 0.51~0.92; maternal: *aOR* = 0.69, 95%*CI* = 0.51~0.92), and paternal psychological control (*aOR* = 1.17, 95%*CI* = 1.00~1.36) was positively associated with pornography use. Maternal connectedness and psychological control were not associated with students' sexual-related behaviors (*P* > 0.05). Person-centered analysis also shows gender differences in associations between parenting style and sexual-related behaviors. Compared with authoritative parenting, free-range and tiger parenting were positively associated with romantic relationships (free-range: *aOR* = 1.65, 95%*CI* = 1.05~2.60; tiger: *aOR* = 1.71, 95%*CI* = 1.01~2.89) and intimate behaviors (free-range: *aOR* = 1.82, 95%*CI* = 1.24~2.66; tiger: *aOR* = 1.79, 95%*CI* = 1.21~2.64) in girls; whereas free-range (*aOR* = 3.93, 95%*CI* = 1.52~10.15), monitoring (*aOR* = 3.25, 95%*CI* = 1.20~8.77), psychological control (*aOR* = 3.69, 95%*CI* = 1.33~10.28) and tiger parenting (*aOR* = 4.47, 95%*CI* = 1.39~14.39) were positively associated with pornography use in boys.

**Conclusions:**

Parental-child connectedness, behavioral monitoring and authoritative parenting style are negatively associated with sexual-related behaviors among secondary vocational school students with some gender differences. It is necessary to develop school-based interventions to encourage authoritative parenting styles and improve gender-specific parenting of secondary vocational school students with emphasis on behavioral monitoring for boys and parent-child connectedness for girl, and calls for advocacy of paternal engagement in parenting, especially for girls.

## 1 Introduction

Sexual and reproductive health (SRH) is fundamental to human health, survival, sustainable economic and social development, and overall wellbeing. Adolescent sexual and reproductive health is a global concern with profound implications for their future wellbeing (Liang et al., 2019). Sexual activity of adolescents varies markedly by gender and region, country. Overall, 14.2% (95% confidence interval: 12.1-16.2) of adolescents aged 12-15 years had sexual intercourse, with boys reporting much higher than girls (19.7%, 16.9-22.5 vs. 8.9%, 7.6-10.3). The prevalence was the highest in the region of the Americas (18.4%, 15.2-21.5) and the lowest in the South-east Asia region (5.3%, 2.6-8.0). Adolescents from high-income and lower middle-income countries had the highest (19.5%, 13.5-25.5) and the lowest (7.3%, 5.5-9.0) prevalence, respectively. Adolescents aged 14-15 years had much higher odds of having sexual intercourse than adolescents aged 12-13 years (*aOR* 1.72, 1.64-1.80) (Kushal et al., 2022). As of 2019, adolescents aged 15–19 years in low- and middle-income countries had an estimated 21 million pregnancies each year, of which approximately 50% were unintended and which resulted in an estimated 12 million births (World Health Organization, 2024). According to the World Health Organization (WHO), 610,000 youth aged 15-24 years were newly infected with HIV in 2016 alone. AIDS is the leading cause of death among adolescents in sub-Saharan Africa and second leading cause for adolescents worldwide (Hosek and Pettifor, 2019).

Young people aged 10 to 24 years in China, who make up about 17% of the population in the country, are facing SRH challenges (NBSPRC, 2023). With socio-economic advancements, adolescents reach sexual maturity at a younger age but their psychological development lags (Song, 2023). Premarital sexual behaviors were not approved in traditional Chinese society, however, the rapid growth of the internet and globalization are transforming the social environments as well as young people's perspectives and attitudes toward premarital sexual behaviors (Li and Liu, 2004). Premarital sex has been accepted by many young people and premarital sexual behaviors, unwanted pregnancy, induced abortion, and repeated abortion among young unmarried people are increasing (Xiao et al., 2022; Yin, 2023). According to the first National Youth Reproductive Health Survey in 2009, 22.4% of unmarried youth aged 15-24 years reported having had sexual intercourse. Adolescents who were older, in higher grades, male, living in developed, coastal, and urban cities were more likely to have sexual intercourse than their counterparts (Zhou et al., 2021). Among adolescents aged 12-14 in Shanghai, the most developed coastal city in China, only 0.9% reported having had sexual intercourse, which was significantly lower than that in other South-east Asia countries (Yu et al., 2021). Although Chinese young people reported low sexual intercourse, their contraceptive use was also low, putting them at risk of unwanted pregnancy, induced abortion and HIV infection. Between 1996 and 2016 in China, induced abortion rate among adolescent girls aged 15-19 years increased from 2.06‰ to 2.76‰,which was lower than that of American counterparts (9.2‰-6.2%‰ between 2012-2016) (Kortsmit et al., 2023; Wang et al., 2023). It has been reported that over 50% of induced abortions in China were performed on women under the age of 25, and the repeated abortion rate exceeds 30% among unmarried women (CFPA, 2020). In addition, between 2010 and 2019, the number of HIV diagnoses among youth aged 15–24 was 9,373 and 15,790, respectively, with an average annual increase of 6.0% (Zhao et al., 2020).

Although romantic relationships, sexual behaviors and pornography use are often regarded as common pathways in adolescent sexual development and exploration in Western countries, abstinence among adolescents is encouraged, and adolescent romantic relationships and sexual-related behaviors are considered deviant behaviors and opposed by families and schools in China (Jin et al., 2021). Research data from China indicate that approximately 28.3%-41.7% of adolescents aged 15-19 have engaged in romantic relationships (Wang et al., 2025; Zhang et al., 2013), 19.4%-31.3% have had intimate sexual behaviors (Liang et al., 2008; Zhang et al., 2013), while 2.4-10.9% have ever had sexual intercourse (Liang et al., 2008; Nie et al., 2022; Zhao et al., 2019). Both parents and school teachers generally avoid discussing sex-related issues directly with adolescents, and thus use of pornographic materials becomes a source of information about sexuality for adolescents to satisfy their curiosity about nude bodies and sexual acts and to arouse themselves (Mattebo et al., 2018; Pirrone et al., 2022). The development of most sexual behaviors is a gradual process. Longitudinal studies on adolescent romantic relationship and sexual trajectories demonstrates that most adolescents followed a progressive sexual trajectory from less intimate (e.g., holding hands, kissing) to more intimate behavior (e.g., sexual intercourse), with the use of pornography having the potential to accelerate this process (Jakobsen, 1997; Pirrone et al., 2022). Therefore, it is important that research on sexual behaviors in adolescents not only examine sexual intercourse as a single outcome but also a broader range of sexual-related behaviors including romantic relationships, kissing, caressing, fondling, petting and pornography use.

Secondary vocational school students represent a specific group in the Chinese education system. Most of them enroll in the schools due to their poor performance in senior high school entrance examinations. They usually enter directly into the labor market after receiving 3 year vocational/technical curriculums. Compared to their senior high school counterparts who continue to pursue tertiary education, secondary vocational students have a higher prevalence of smoking (35.60% vs. 19.29%), drinking (23.60% vs. 14.47%) (Wang et al., 2012), romantic relationships (38.3% vs. 28.3%) and sexual behaviors (18.1% vs. 7.3%) (Zhang et al., 2013). HIV/AIDS surveillance data from Tianjin city, China indicated that only 49.3% of secondary vocational school students used condoms during their first sexual intercourse, with a mere 16.4% reporting consistent use (Liu et al., 2017).

Based on Bronfenbrenner's ecological systems theory (Bronfenbrenner, 1986), family as a microsystem directly affects individual development. Considerable research has been documented that parents have tremendous influence on adolescent behavior problem (Barber et al., 2005) as well as sexual behaviors and health outcomes (Xu, 2018). Previous studies have demonstrated that parenting practices are closely linked to adolescents‘ sexual-related behaviors and healthy development (van de Bongardt et al., 2014; Wang et al., 2015), which have implications for programs aiming to prevent adolescent risky sexual behaviors. Parenting practices refer to the behaviors exhibited by parents in specific contexts of parent-child interactions (Wood et al., 2003). Although parenting is a multidimensional and complex construct, researchers have reached a consensus on at least two fundamental dimensions, namely parental support and parental control, with the latter further subdivided into psychological control and behavioral control (Power, 2013). The support is a dimension of the parent-adolescent relationship that is otherwise called warmth, affection, care, comfort, concern, nurturance, connection or love (World Health Organization, 2007). Parenting style refers to a stable pattern or tendency of parenting practices (Baumrind, 1967). Based on two dimensions of parenting practices: responsiveness (parental acceptance and responsiveness, along with sensitivity to children's needs) and demandingness (the extent to which parents demand compliance with set standards via supervision and discipline). Baumrind (1967) and Maccoby and Martin (1983) proposed a parenting style model with four parenting subtypes: authoritative parenting (high responsiveness and high demandingness), authoritarian parenting (low responsiveness and high demandingness), permissive parenting (high responsiveness and low demandingness) and neglecting parenting (low responsiveness and low demandingness) (Abdul Gafor and Kurukkan, 2014; Baumrind, 1971). Self-determination theory posits that individual behavior is largely driven by innate psychological needs. The fulfillment of three basic psychological needs, which are autonomy, competence, and relatedness, serves to enhance internal motivation, facilitate the internalization of external motivation, and support healthy development. In family contexts, high levels of parental-child connectedness meet adolescents' need for relatedness, fostering a sense of belonging. Low levels of psychological control allow adolescents to experience psychological freedom, satisfying their need for autonomy. Parental behavioral monitoring establishes appropriate rules and conveys expectations and guidance for conduct, providing structure that enhances adolescents‘ sense of mastery and efficacy, thereby fulfilling their need for competence (Ulferts, 2020). Among the four parenting styles, authoritative parenting stands out as the most conducive to positive developmental outcomes. Authoritative parenting's balance of warmth and firm guidance provides a secure foundation for adolescents to develop autonomy while still feeling supported. In contrast, authoritarian parenting, with its emphasis on control and lack of warmth, tends to stifle emotional expression and encourages compliance rather than independent thinking, which can negatively affect adolescent self-esteem and social relationships. Permissive parenting, although characterized by warmth and acceptance, often lacks the structure and discipline necessary for healthy adolescent development. Adolescents with permissive parenting may struggle with boundaries, self-control, and responsibility, which can manifest in impulsivity and poor decision-making. Neglectful parenting, defined by a lack of emotional engagement and supervision, tends to result in the most problematic outcomes. Neglected adolescents often experience attachment issues, lower self-esteem, and increased susceptibility to risky behaviors, such as substance abuse or delinquency (Molanos, 2025). Researches have consistently shown that parent-child connectedness (*B* = −0.39, *p* < 0.05) and parental monitoring (β = −0.118; *p* < 0.01) have positive impact (Sieverding et al., 2005; van de Bongardt et al., 2014) while psychological control (mother: *t* = 2.27, *p* = 0.02; father: *t* = 3.89, *p* < 0.001) has negative impact on adolescents' sexual risk behaviors (Oudekerk et al., 2014).

Recent research evidence suggests that the parental impact may vary by cultural contexts and the gender of both adolescent and their parents (Fung and Lau, 2012; Yaffe, 2023a). For example, Western cultures prioritize autonomy, self-confidence, and independence as key goals in children's development, while Chinese cultures value interdependence, emphasizing self-restraint and adaptability to social norms (Fung and Lau, 2012). In Western values, parental strictness may be interpreted by children as hostility, aggression, or distrust, whereas Chinese children may perceive it as care and high expectations (Xu et al., 2013). In addition, study suggests that mothers and fathers play a distinct role in child-rearing in the family, with mothers being more present and more accepting, responsive, supportive, behaviorally controlling, and autonomy granting than fathers (Yaffe, 2023b). The gender of the adolescents also affects parenting behaviors. In China, parents often provide more support and supervision to girls and have higher expectations for boys (Yang, 2025). However, few studies have been conducted to explore the links between parenting and adolescents' sexual-related behaviors in Chinese culture and its gender difference.

While some studies have examined the association between parenting practice and adolescent sexual-related behaviors, many of them focus on a particular dimension (known as variable-centered approach) rather than exploring the role of parenting practice by integrating all the dimensions into a composite variable (Oudekerk et al., 2014; Sneed et al., 2009). Variable-centered research approaches aim to examine the relationships among variables, investigating which factors account for the magnitude of variance in the dependent variable (Yin et al., 2020). Although a variable-centered modeling approach has the advantage of evaluating the influence of each parenting dimension individually using all available data (Power, 2013), its limitation cannot be ignored. It is important to note that parenting dimensions often interact in combinations to impact adolescent behaviors and health outcomes (Mandara, 2003). Person-centered approaches treat variables as an interdependent system, using multiple characteristics to classify participants into distinct subgroups and then examining their antecedents and consequences (Yin et al., 2020). As an alternative way, this approach can be utilized to identify subgroups of individual based on their similarity in the scores on a set of parenting dimension variables, which allows for the comparison between subgroups (Stanley et al., 2017). Previous evidence suggested that adolescent-perceived parenting practices were more representative of their personal experiences than parent-reports (Van Lissa et al., 2019). The study aims to explore the gender-specific associations between perceived paternal and maternal parenting practice and sexual-related behaviors among secondary vocational school students in China using both variable-centered and person-centered modeling approaches. Based on Bronfenbrenner's ecological systems theory, self-determination theory, and previous research, we hypothesized that: H1: Perceived parenting behaviors or styles characterized by high level of connectedness and behavioral control, but lower level of psychological control would be associated with a lower risk of engaging in sexual-related behaviors among secondary vocational students; H2: These associations vary by gender.

## 2 Methods

### 2.1 Participants and procedures

The cross-sectional study was conducted in Shanghai Municipality and Shaanxi Province in China from April to June in 2021. Shanghai Municipality, a province-level administrative unit, is the most developed and open coastal city in Eastern China with a blend culture of East and West and a permanent population of 24.87 million, and Shaanxi Province is a typically less-developed province in the inland regions of Northwest China with more traditional cultural values and a permanent population of 39.53 million. Using convenience sampling method, six secondary vocational schools were chosen with one from each of the core district, inner suburbs, and outer suburbs of Shanghai Municipality, as well as one from each of the three cities with high, medium, and low economic level in Shaanxi Province. Cluster sampling was used to select 500-600 students in each school with an equal proportion of gender and grade. A total of 3,237 students participated in the survey. After excluding 100 who provided invalid information because of duplicate submissions or patterned responses (e.g., straight-lining) and 745 cases who did not live with both parents for most of their lifetime, 2,392 students were included in the study. The sample size for this study was estimated using the following formula:


$$\begin{array}{l} n=\frac{Z_{1-\alpha /2}^{2}\;*\;p\left(1-p\right)}{d^{2}}\;*\;DE \end{array}$$

Based on previous data, the anticipated prevalence (*p*) of romantic relationships among secondary vocational students is 42% (Wang et al., 2025); the margin of error (*d*) is set at 0.1*p*; the significance level (α) is 0.05 (two-tailed), *z*-value = 1.96; the design effect for cluster sampling is 1.5; anticipated non-response rate is 5%; the required sample size is approximately 838.

The survey was conducted via an anonymous online self-administered electronic questionnaire. Logical checks and skip patterns were incorporated during its design to improve data accuracy and completeness. The questionnaire was revised and finalized following a discussion with school teachers and a pilot testing among male and female students. Two trained investigators presented at each school to facilitate the survey process. Prior to distributing the questionnaires, investigators emphasized the significance and confidentiality of the study to participants. School teachers arranged students with spaced seating to prevent communication. After completing the questionnaires, students retained screenshots for verification with the backend system. The study was approved by the institutional review board of Shanghai Institute of Planned Parenthood Research (NO. PJ2021-24). Parental consent and participants' assent were collected before data collection.

### 2.2 Measures

#### 2.2.1 Demographic characteristics

Demographic characteristics include study site (Shanghai/Shaanxi), gender, grade, local resident or not, only child in the family or not, perceived family economic status (good/average/poor), parental relationship (good/average/poor), parental education (junior high and below/senior high/college and above), perceived peers' romantic relationships (none of them/few/about half/most/all/unknown).

#### 2.2.2 Parenting practices

Three parenting dimensions for both father and mother were measured from the perspective of adolescents in the study, including parental-child connectedness, behavior monitoring, and psychological control. All instruments were translated and adapted for the local context and validated by experts.

*Parental-child connectedness* refers to parents' emotional warmth and support toward their child. This dimension was measured by a four-item scale developed by Sidze et al. (e.g., “how often their parents/guardians encouraged them”) (Sidze et al., 2015). Participants answered questions on a 4-point scale ranging from 1 (never) to 4 (always). A higher mean score indicates a higher level of perceived connectedness with a particular parent. Cronbach's alphas were 0.88 for fathers and 0.88 for mothers.

*Behavior monitoring* refers to parents' awareness of their children's behavior and their efforts to prevent risky behavior by monitoring and guiding their actions (Sieverding et al., 2005). This dimension was measured by an eight-item scale developed by Wang et al. (e.g., “when I get home from school, I have to get their permission before I go out again”) (Wang et al., 2007). Participants answered questions on a 5-point scale ranging from 1 (never) to 5 (always). A higher mean score indicates more perceived maternal/paternal behavior monitoring. Cronbach's alphas were 0.92 for fathers and 0.91 for mothers.

*Psychological control* refers to parents trying to control the children's thoughts or feelings through guilt induction, love withdrawal, shaming, and other disrespectful or manipulative behaviors (Romm et al., 2020). This dimension was measured with three items: my mother/father is a person who (1) is always trying to change how I feel or think about things, (2) brings up past mistakes when she/he criticizes me, and (3) is less friendly with me if I do not see things her/his way. Participants answered questions on a 5-point scale ranging from 1 (not like her/him at all) to 5 (exactly like her/him at all) (Barber et al., 2005). A higher mean score indicates more perceived maternal/paternal psychological control. Cronbach's alphas were 0.86 for fathers and 0.86 for mothers.

*Parenting style* Based on the above-mentioned three dimensions, five subtypes of parenting style which are labeled as behavioral monitoring parenting, free-range parenting, psychological control parenting, tiger parenting and authoritative parenting were identified through latent profile analysis which described in detail elsewhere (Zheng et al., 2022). *Behavioral monitoring parenting* is characterized by a moderate to high level of parental-child connectedness and behavior monitoring, and a moderate level of psychological control. *Free-range parenting* has a moderate level of parental-child connectedness, low levels of behavior monitoring and psychological control.

*Psychological control parenting* includes a low level of parental-child connectedness and behavior monitoring, and a high level of psychological control. *Tiger parenting* features a moderate level of parental-child connectedness, and the highest levels of behavior monitoring and psychological control. *Authoritative parenting* entails a high level of parental-child connectedness, moderate to high levels of behavior monitoring, and the lowest level of psychological control.

#### 2.2.3 Sexual-related behaviors

Life-time romantic relationships, intimate behaviors, sexual intercourse, and pornography use were measured by asking four Yes/No questions: have you ever (1) had a romantic relationship, (2) engaged in an intimate behavior (e.g., kissing, caressing, fondling, petting), (3) had sexual intercourse with opposite sex, and (4) purposely used any forms of pornography (e.g., pictures, electronic images, audio porn, videos, written material).

### 2.3 Statistical analysis

*T*-tests were used to compare the mean of parenting dimensions between parents and by gender of students. Chi-square tests were used to compare gender differences in parenting style and sexual-related behaviors. For multivariable analysis, a multi-level null model was performed to detect the clustering of behavioral outcome variables across school and class levels. The findings indicated no clustering at the school level, but clustering at the class level was observed. Spearman correlation analysis (*r* = − 0.48-0.71) and variance inflation factor (VIF) (VIF = 1.03-2.22, mean VIF = 1.5) were conducted to check multicollinearity diagnostics between independent variables. Finally, a two-level multivariable logistic regression model was used with controlling for clustering at the class level to examine the associations between perceived paternal and maternal parenting practices and students' sexual-related behaviors, as well as the gender differences in this association. The covariates adjusted in the model included study site, only child in the family or not, grade, a local resident or not, family economic status, parental relationship, parental education, and perceived peers' romantic relationships. The differences in the effects of paternal and maternal parenting dimensions on students' sexual-related behaviors were examined using the seemingly unrelated regression model. The significance level was set at α = 0.05, with statistical significance determined when the two-tailed test yielded a *P*-value < 0.05. Data were analyzed by using Stata/SE 15.1 (StataCorp LLC, College Station, TX, USA).

## 3 Results

### 3.1 Socio-demographic characteristics of participants

The sample included 1,121 boys (46.86%) and 1,271 girls (53.14%), with an average age of 16.93 ± 0.03 years. Only-child made up 41.51% of the sample. Among the participants, 50.84% were from Shanghai Municipality and 49.16% from Shaanxi Province. A majority (81.31%) of the participants were local residents. In terms of family background, 64.84% reported an average family economic status and 79.22% perceived good relationship between their parents. More than half of their mothers (62.02%) and fathers (55.99%) had junior high and below education.

### 3.2 Scores of parenting dimensions

Compared with fathers, mothers scored higher in parent-child connectedness and behavior monitoring for both boys and girls, and in psychological control for girls. There was no significant difference in psychological control for boys between parents. Compared with girls, fathers scored higher in parent-child connectedness and psychological control for boys, while both fathers and mothers scored lower in behavior monitoring for boys. The detailed results are shown in Table 1.

**Table 1:** Mean comparison of parenting dimensions (Mean ± SD, *n* = 2392).

**Variable**	**Connectedness**		**Behavior monitoring**		**Psychological control**	
	**Paternal**	**Maternal**	**Paternal**	**Maternal**	**Paternal**	**Maternal**
Boys	3.17 ± 0.96^**^	3.33 ± 0.96^###^	2.97 ± 1.05^*^	3.06 ± 1.03^***#^	2.72 ± 1.09^**^	2.72 ± 1.07
Girls	3.04 ± 0.99	3.26 ± 0.97^###^	3.06 ± 1.02	3.20 ± 0.97^###^	2.58 ± 1.05	2.67 ± 1.06^#^
Total	3.10 ± 0.98	3.29 ± 0.97^###^	3.02 ± 1.03	3.13 ± 1.00^###^	2.65 ± 1.07	2.69 ± 1.06

Differences between boys and girls: ^*^P < 0.05, ^**^P < 0.01, ^***^P < 0.001; Differences between fathers and mothers: ^#^*P* < 0.05, ^*##*^*P* < 0.01, ^*###*^*P* < 0.001.

### 3.3 Distribution of parenting style

As shown in Table 2, behavioral monitoring parenting and free-range parenting comprised more than 60% of the sample with authoritative parenting being the least subtype (11.75%). Compared with boys, girls had more authoritative parenting but less psychological control parenting.

**Table 2:** Distribution of parenting style, by gender (%).

**Subtype**	**Total (*n* = 2392)**	**Boys (*n* = 1121)**	**Girls (*n* = 1271)**	***χ^2^***	***P***
Monitoring	33.65	32.47	34.70		
Free-range	27.05	28.19	26.04	14.40	0.006
Psychological control	14.38	16.06	12.90		
Tiger	13.17	13.65	12.75		
Authoritative	11.75	9.63	13.61		

### 3.4 The prevalence of sexual-related behaviors

Tables 3 shows that approximately 49% of the students had ever had romantic relationships, 32% had engaged in intimate behaviors, 5% had engaged in sexual intercourse, and 10% had pornography use. Boys were significantly more likely to have these sexual-related behaviors than girls (*P* < 0.05).

**Table 3:** Prevalence of sexual-related behaviors, by gender (*n* (%)).

**Behavior**	**Total (*n* = 2392)**	**Boys (*n* = 1121)**	**Girls (*n* = 1271)**	**χ^2^**	***P***
Romantic relationships	1172(49.00)	597(53.26)	575(45.24)	15.32	< 0.001
Intimate behaviors	755(31.56)	392(34.97)	363(28.56)	11.33	0.001
Sexual intercourse	116(4.85)	85(7.58)	31(2.44)	34.15	< 0.001
Pornography use	232(9.70)	166(14.81)	66(5.19)	62.88	< 0.001

### 3.5 The association between parenting dimensions and sexual-related behaviors

Table 4 indicates that parental connectedness, behavior monitoring and psychological control were associated students' sexual-related behaviors with gender differences. Specifically, perceived paternal connectedness was negatively associated with romantic relationships (*aOR* = 0.82, 95%*CI* = 0.71~0.94) and intimate behaviors (*aOR* = 0.83, 95%*CI* = 0.71~0.97) among girls only. No significant association was observed between perceived maternal connectedness and any sexual-related behaviors in both boys and girls. Both perceived paternal and maternal behavior monitoring were negatively associated with intimate behaviors (paternal: *aOR* = 0.86, 95%*CI* = 0.75~0.98; maternal: *aOR* = 0.83, 95%*CI* = 0.73~0.95) and sexual intercourse (paternal: *aOR* = 0.69, 95%*CI* = 0.51~0.92; maternal: *aOR* = 0.69, 95%*CI* = 0.51~0.92) among boys only. No significant association was found between paternal and maternal behavior monitoring and romantic relationships or pornography use in both boys and girls. Paternal psychological control was positively associated with pornography use among boys only (*aOR* = 1.17, 95%*CI* = 1.00~1.36). No significant association was observed between paternal psychological control and other sexual-related behaviors, nor between maternal psychological control and any sexual-related behaviors. The results of the seemingly unrelated regression models showed that there were significant differences between the effects of paternal and maternal connectedness on students' romantic relationships, girls' sexual intercourse and pornography use (all *p* < 0.05).

**Table 4:** Relationship between parenting dimensions and sexual-related behaviors, by gender (*aOR*(95%*CI*)).

**Variables**	**Total (*n* = 2167)**	**Boys (*n* = 1013)**	**Girls (*n* = 1154)**
**Romantic relationships**			
Connectedness (Maternal)	0.95 (0.84,1.08)^#^	1.00 (0.83,1.20)	0.92 (0.78,1.07)^#^
Connectedness (Paternal)	0.88 (0.78,1.00)^*^	0.95 (0.80,1.14)	0.82 (0.71,0.94)^**^
Behavior monitoring (Maternal)	0.91 (0.82,1.01)	0.92 (0.79,1.06)	0.91 (0.80,1.03)
Behavior monitoring (Paternal)	0.93 (0.84,1.03)	0.95 (0.82,1.10)	0.91 (0.82,1.02)
Psychological control (Maternal)	1.02 (0.94,1.11)	1.00 (0.88,1.14)	1.04 (0.93,1.16)
Psychological control (Paternal)	1.05 (0.97,1.14)	1.04 (0.90,1.19)	1.08 (0.97,1.19)
**Intimate behaviors**			
Connectedness (Maternal)	0.91 (0.81,1.02)	0.94 (0.81,1.10)	0.87 (0.75,1.01)
Connectedness (Paternal)	0.87 (0.77,0.98)^*^	0.91 (0.80,1.04)	0.83 (0.71,0.97)^*^
Behavior monitoring (Maternal)	0.87 (0.79,0.97)^**^	0.83 (0.73,0.95)^**^	0.92 (0.79,1.06)
Behavior monitoring (Paternal)	0.88 (0.80,0.97)^**^	0.86 (0.75,0.98)^*^	0.90 (0.80,1.02)
Psychological control (Maternal)	1.06 (0.97,1.15)	1.07 (0.96,1.19)	1.04 (0.92,1.17)
Psychological control (Paternal)	1.08 (0.98,1.18)	1.10 (1.00,1.21)	1.05 (0.92,1.21)
**Sexual intercourse**			
Connectedness (Maternal)	0.87 (0.71,1.06)	0.85 (0.67,1.09)	0.92 (0.58,1.45)^#^
Connectedness (Paternal)	0.83 (0.66,1.06)	0.89 (0.70,1.14)	0.71 (0.41,1.22)
Behavior monitoring (Maternal)	0.73 (0.57,0.93)^**^	0.69 (0.51,0.92)^*^	0.85 (0.55,1.31)
Behavior monitoring (Paternal)	0.76 (0.60,0.96)^*^	0.69 (0.51,0.92)^*^	0.96 (0.58,1.59)
Psychological control (Maternal)	1.06 (0.85,1.31)	1.06 (0.82,1.36)	1.04 (0.72,1.49)
Psychological control (Paternal)	1.12 (0.92,1.36)	1.08 (0.85,1.37)	1.22 (0.88,1.70)
**Pornography use**			
Connectedness (Maternal)	0.94 (0.77,1.14)	0.88 (0.70,1.10)	1.08 (0.74,1.59)^#^
Connectedness (Paternal)	0.80 (066,0.96)^*^	0.80 (0.65,1.00)	0.75 (0.54,1.05)
Behavior monitoring (Maternal)	0.87 (0.75,1.01)	0.84 (0.69,1.03)	0.92 (0.69,1.21)
Behavior monitoring (Paternal)	0.89 (0.77,1.02)	0.89 (0.73,1.07)	0.87 (0.69,1.10)
Psychological control (Maternal)	1.09 (0.95,1.24)	1.11 (0.94,1.30)	1.02 (0.78,1.34)
Psychological control (Paternal)	1.13 (1.01,1.28)^*^	1.17 (1.00,1.36)^*^	1.05 (0.82,1.36)

Association between parenting behaviors and sexual-related behaviors: ^*^P < 0.05, ^**^P < 0.01; Differences between fathers and mothers: ^#^*P* < 0.05

### 3.6 The association between parenting style and sexual-related behaviors

As shown in Table 5, parenting style was significantly associated with romantic relationships, intimate behaviors and pornography use with gender differences. Compared with authoritative parenting, free-range parenting was significantly associated with girls' romantic relationships (*aOR* = 1.65, 95%*CI* = 1.05~2.60) and intimate behaviors (*aOR* = 1.82, 95%*CI* = 1.24~2.66) and boys' pornography use (*aOR* = 3.93, 95%*CI* = 1.52~10.15); monitoring parenting was significantly associated with boys' pornography use (*aOR* = 3.25, 95%*CI* = 1.20~8.77); psychological control parenting was significantly associated with boys' pornography use (*aOR* = 3.69, 95%*CI* = 1.33~10.28); and tiger parenting was significantly associated with girls' romantic relationships (*aOR* = 1.71, 95%*CI* = 1.01~2.89) and intimate behaviors (*aOR* = 1.79, 95%*CI* = 1.21~2.64) and boys' pornography use (*aOR* = 4.47, 95%*CI* = 1.39~14.39).

**Table 5:** Relationship between parenting style and sexual-related behaviors, by gender (*aOR*(95%*CI*)).

**Variables**	**Total (*n* = 2167)**	**Boys (*n* = 1013)**	**Girls (*n* = 1154)**
**Romantic relationships**			
Authoritative	1.00	1.00	1.00
Free-range	1.32 (0.93,1.87)	1.03 (0.59,1.81)	1.65 (1.05,2.60)^*^
Monitoring	1.11 (0.81,1.51)	0.98 (0.58,1.64)	1.22 (0.80,1.88)
Psychological control	1.24 (0.89,1.72)	1.07 (0.68,1.70)	1.35 (0.81,2.24)
Tiger	1.39 (1.00,1.92)^*^	1.09 (0.66,1.81)	1.71 (1.01,2.89)^*^
**Intimate behaviors**			
Authoritative	1.00	1.00	1.00
Free-range	1.58 (1.14,2.21)^**^	1.41 (0.82,2.42)	1.82 (1.24,2.66)^**^
Monitoring	1.16 (0.87,1.54)	1.00 (0.64,1.58)	1.30 (0.92,1.84)
Psychological control	1.42 (1.02,1.99)^*^	1.49 (0.90,2.47)	1.29 (0.78,2.11)
Tiger	1.68 (1.21,2.32)^**^	1.59 (0.99,2.55)	1.79 (1.21,2.64)^**^
**Sexual intercourse**			
Authoritative	1.00	1.00	1.00
Free-range	1.30 (0.75,2.24)	1.40 (0.68,2.88)	1.13 (0.23,5.55)
Monitoring	0.67 (0.29,1.57)	0.69 (0.25,1.92)	0.70 (0.15,3.26)
Psychological control	1.36 (0.65,2.85)	1.09 (0.43,2.77)	2.22 (0.45,10.91)
Tiger	1.21 (0.53,2.78)	1.24 (0.42,3.66)	1.36 (0.49,3.80)
**Pornography use**			
Authoritative	1.00	1.00	1.00
Free-range	2.94 (1.44,5.99)^**^	3.93 (1.52,10.15)^**^	1.76 (0.63,4.87)
Monitoring	2.23 (1.06,4.68)^*^	3.25 (1.20,8.77)^*^	1.01 (0.31,3.30)
Psychological control	2.11 (0.96,4.63)	3.69 (1.33,10.28)^*^	0.49 (0.12,2.11)
Tiger	3.49 (1.51,8.07)^**^	4.47 (1.39,14.39)^*^	1.82 (0.58,5.73)

^*^P < 0.05, ^**^ *P* < 0.01. The ‘Authoritative' parenting style serves as the reference group.

## 4 Discussion

This study explored the gender-specific association between perceived maternal and paternal parenting practices and sexual-related behaviors among secondary vocational school students by using both variable-centered and person-centered modeling approach in Chinese culture. The finding from variable-centered modeling approach shows that fathers play a crucial role for their children's development of sexual-related behaviors, especially for girls. In addition, parental-child connectedness was more salient for the development of sexual-related behaviors for girls than boys, whereas behavior monitoring and psychological control had more impact on sexual-related behaviors for boys than girls. The finding from person-centered modeling approach shows that authoritative parenting with a delicate balance between the dimensions was the most beneficial subtype of parenting for the development of sexual-related behaviors for both boys and girls. To the best of our knowledge, this is the first study to explore the relationship between parenting practice and sexual-related behaviors in China.

Overall, the perceived parenting dimensions among secondary vocational school students in this study are generally in line with previous studies conducted among high school students in China (Li, 2013; Zhang, 2019). With regards to paternal and maternal differences in perceived parenting, the current results are consistent with previous research in China that mothers scored higher than fathers in both parent-child connectedness and behavior monitoring dimensions, indicating more investment of mothers in comparison to fathers in China (Deng et al., 2018; Li, 2013). In traditional Chinese parental roles, fathers are stricter, while mothers give more love and care to their children (Shek D. T. L., 2008). This finding suggests that the traditional norms about family role division that “men outside the home, women inside” continues to influence Chinese parents‘ investments in raising children. In terms of gender differences in perceived parenting, boys in this study perceived stronger father-child connectedness and psychological control but weaker paternal and maternal behavior monitoring than girls, which is basically consistent with previous studies in China (Li, 2017; Zhang, 2019, 2012). In a traditional patriarchal society in China, men are the head of the family and a preference for sons over daughters is deeply rooted (Chen, 2023). Families and society usually have high expectations for boys. Due to the impact of the traditional values, some fathers may give their sons more support and love than their daughters, leading to stronger paternal connectedness for boys than girls. The reason for stronger paternal psychological control for boys than girls might be related to high expectations for sons on the one hand (Shek D. T., 2008), and the differences in personality traits, communication styles, and behavior characteristics between boys and girls on the other hand. Boys are more adventurous, more eager for independence, and prone to conflict with parents, which may trigger parental psychological control (Feldman et al., 1999). Perceived stronger parents' behavior monitoring for girls reflects a stronger sense of protection for girls by parents (Shek D. T. L., 2008). The gender differences in perceived parenting dimensions also lead to gender differences in subtypes of parenting style, i.e., more psychologically controlled parenting for boys and more authoritative parenting for girls.

Previous studies suggest that a good parent-child relationship can delay the onset of sexual activity and reduce risky sexual behaviors among adolescents (van de Bongardt et al., 2014). A study by Gillmore suggests that a mother's emotional warmth helps to form a positive and stable interaction between parent and child, and parent-child communication based on this interaction plays a guiding and protective role in children's behavioral decisions (Gillmore et al., 2011). In addition, according to developmental and ecological theory, poor parent-child relationships make adolescents more susceptible to adverse peer pressure and norms (Allen et al., 2002). There are two possible reasons for this: first, if adolescents lack the warm support from adults, they may have psychological and social adaptation difficulties or low self-esteem (Ephross, 1980); second, adolescents who lack a strong relationship with their caregivers may be more eager to establish relationships with others (such as peer relationships and sexual intimate relationships) to meet their need for interpersonal recognition (Cross and Madson, 1997). In this study, father-child connectedness is negatively associated with students' sexual-related behaviors, which is consistent with previous research (Blum et al., 2003). In contrast to previous studies (Miller et al., 1997), this study did not find a significant association between mother-child connectedness and sexual-related behaviors. This is possibly attributed to the generally high perceived mother-child connectedness in this study. This result suggests that father-child connectedness may play a more crucial role than mother-child connectedness.

Accumulating evidence has indicated that parental behavior monitoring is associated with decreased sexual behaviors and later age at sexual debut (Wight et al., 2006). Our study also found that both paternal and maternal behavior monitoring were negatively associated with students' intimate behaviors and sexual intercourse. Research indicates that parental behavior monitoring can facilitate the enhancement of adolescents‘ self-control abilities (Deng et al., 2018), reduce the negative influence of peers (Wang et al., 2015) and limit their opportunity to engage in sexual-related behaviors (Kapungu et al., 2006). In contrast to previous research conducted among junior or senior high school students (Nieh et al., 2020), a negative association between parental behavior monitoring and pornography use was not observed in this study. The reason may be that secondary vocational school students have lower academic pressure than junior and senior high school students and thus their parents might have less control over their online behaviors.

There has been clear evidence that parental psychological control is associated with adolescent internalizing and externalizing problems (Finkenauer et al., 2005). Parental psychological control hinders the development of adolescents‘ self-esteem and autonomy (Liu, 2020). Adolescents who fail to establish autonomy in relationships with parents are unlikely to demonstrate autonomy in peer relationships and are more susceptible to the influence of peer's values and norms (Allen et al., 2012). In this study, fathers' psychological control was positively associated with pornography use, which is consistent with previous research (Nieh et al., 2020). No association was found between paternal psychological control and other sexual behaviors, nor any association was found between maternal psychological control and sexual behaviors, which is contrary to previous research in Western culture (Oudekerk et al., 2014). This may be because most of the tools for measuring psychological control were developed in a Western cultural context, and intrusive parenting behaviors in Western culture may have different meanings in other cultural contexts. For example, the use of shaming is viewed as negative in Western culture, while it is one of the ways to promote children's moral socialization in China (Helwig et al., 2014). Chinese parenting emphases on reflection, bringing honor to the family and unconditional obedience (Shek D. T. L., 2008). When children perceive a parenting practice to be culturally normative or beneficial to them, the negative association is often mitigated (Helwig et al., 2014). A study comparing the association between parental psychological control and children's behavior problems among European American and Hong Kong Chinese families found that the association was observed in European American families but not in Hong Kong Chinese families, supporting the “cultural specificity” hypothesis. Whether parental psychological control has the same negative effect in Chinese culture as in Western culture needs further research (Fung and Lau, 2012).

Authoritative parenting is universally recognized as the most beneficial for the healthy growth of adolescents from the perspective of parenting style (Baumrind, 1966), and this study also found similar results through a person-centered approach. Unfortunately, it is less prevalent among parents of secondary vocational students. Possibly due to the low prevalence of sexual intercourse and limited sample size, no significant association was found between parenting style and sexual intercourse in this study. However, a large body of literature demonstrates that positive parenting style can reduce adolescents' risky sexual behaviors including early sexual intercourse, multiple sexual partners, and non-use of condoms (Yimer and Ashebir, 2019). Comparing the scores of the three parenting dimensions in the five subtypes of parenting style in this study, it was found that low parent-child connectedness and behavior monitoring (i.e., free-range parenting), and high psychological control (i.e., tiger and psychological control parenting) are detrimental to the development of sexual-related behaviors in secondary vocational school students, supporting the view that a delicate balance between the dimensions is crucial (Yimer and Ashebir, 2019).

In terms of the gender differences, our study found that father-child connectedness was negatively associated with romantic relationships and intimate behaviors in girls only, while paternal and maternal behavioral monitoring were negatively associated with intimate behaviors and sexual intercourse in boys only. This is consistent with the findings from studies comprised of predominantly ethnic-minority American youth suggesting that parental warmth and emotional connection may be a more important protective factor for girls, whereas monitoring may be more effective in reducing sexual risk behaviors in boys than in girls (Kincaid et al., 2012). Hoffman proposed that due to differences in genetic structure, personality, etc., boys and girls will react differently to different parenting practices (Hoffman, 1977). In general, girls tend to be more interpersonally oriented and have more pronounced attachment needs than boys; thus, girls may be more likely to be affected by deficits in the parent-child connectedness (Gilligan, 1993; Kincaid et al., 2012). Furthermore, for females, sexual intimacy is an internalization process, but for males, whether to establish a sexual relationship is more determined by externalized behavioral factors (Gilligan, 1993). Previous evidence has shown that father absence is associated with early sexual behavior and unintended pregnancy in girls (Ngom et al., 2003). As the first and most important adult male a daughter is exposed to, fathers' behaviors shape their daughters' perceptions of masculinity. Furthermore, paternal support can significantly enhance girls' self-esteem and autonomy, helping them to discourage an unwanted sexual encounter (Bowling and Werner-Wilson, 2000). In the absence of father while growing up, girls may have a strong longing to establish a relationship with the opposite sex as compensation for fatherly love (Mendle et al., 2009; Wang et al., 2017). However, the results of this study suggest that even in two-parent families, a low level of father-daughter connectedness also has a negative impact on the development of girls' sexual-related behaviors. Other research also indicates that the quality of the father-adolescent relationship is much more significant than the amount of time in terms of adolescent development (Sheeber et al., 2007). Significant efforts need to be made to encourage the investment of fathers, especially for girls and support gender equality in caregiving. On the other hand, boys tend to place more value on authority and control from the perspective of social learning (Maccoby, 1990); thus, boys may be more likely to be affected by parental behavior monitoring. Structured, rule-based family environments not only promote boys' development of psychological resilience, but also help them to foster internal self-regulation, thus helping reduce sexual risk behaviors. Studies show that boys tend to exhibit more behavioral problems in less structured settings (Kincaid et al., 2012). In addition, this study found that paternal psychological control was only positively associated with boys' pornography use. High psychological control can undermine father-son relationships and father-son communication about sexual topics (if any), leading to pornography use as an alternative source of sexual information. Additionally, discussing and sharing pornography is more common among boys than girls, and those perceiving high psychological control may be particularly susceptible to peer influence regarding pornography use (Nieh et al., 2020). Previous research indicated that paternal psychological control predicts risky sexual behavior in adolescent boys. Boys may perceive such control as emasculating and demeaning, creating direct conflict with the development of autonomy and self-assertion (Oudekerk et al., 2014). Not surprising, gender difference in the association between parenting style and students' sexual-related behaviors was also observed. Girls reared by subtypes of parenting with low connectedness and low monitoring or high psychological control were more likely to have romantic relationships and intimate behaviors, whereas boys reared by subtypes of parenting with low connectedness and monitoring and high psychological control were more likely to use pornography. In a longitudinal study of African American youth in impoverished communities, boys reared in low control and high warmth (i.e., permissive parenting) homes and girls reared in high control and low warmth (i.e., authoritarian parenting) homes were particularly prone to early intimate behaviors and sexual intercourse, indicating that a high maternal warmth increased the risk of sexual behaviors without the balance of behavior control, and vice versa (Kapungu et al., 2006). Therefore, maintaining a delicate balance between the dimensions of parenting is vitally important for the healthy development of both boys and girls.

There are several limitations that need to be noted. First, this study was conducted among secondary vocational school students from intact families in Shanghai Municipality and Shaanxi Province. The findings might not be generalized to adolescents with other backgrounds, especially those from single-parent families. However, this enables a comparison between fathers and mothers and expands the understanding of father's influence in previous research. Research suggests that children of single parents are at heightened risk of precocious sexual behavior, sexually transmitted diseases, and other risky sexual outcomes (Dufur et al., 2018). Compared with adolescents in intact families, those in non-intact families showed more deviant behaviors and depression in China (Yang and Jiang, 2023). Future research should develop parenting practices measures suitable for non-traditional family structures to enable a more comprehensive understanding of how single-parent family environments influence adolescent sexual outcomes in China. Second, this study used a self-report instrument to collect data. Due to the nature of the questions, especially for sexual-related behaviors, the data were subject to report bias. However, the privacy and confidentiality of participants were protected to reduce the bias in this study. Third, we are not able to explore the interaction between parental parenting dimensions and students' self-esteem, self-control, and self-efficacy in this study, which warrants further research. Finally, this study had the limitations inherent to any cross-sectional study, which cannot determine the causal relationship between parental parenting practices and sexual-related behaviors. Despite these limitations, this study fills in the gaps in previous research by explored the gender-specific associations between paternal and maternal parenting practices and sexual-related behaviors in the Chinese cultural context and provides valuable implications for programs aiming to help parents to prevent adolescent risky sexual behaviors.

## 5 Conclusion

Perceived paternal and maternal parenting practices are closely related to sexual-related behaviors among secondary vocational school students with some gender differences. Fathers play a more important role in children's development of sexual related behaviors than mothers, particularly for girls. In general, parent-child connectedness is more likely to associated with girls' sexual-related behaviors, while parent-child behavior monitoring and psychological control are more likely to associated with boys' sexual-related behaviors. The optimal parenting style is authoritative parenting with high levels of parental-child connectedness, moderate to high levels of behavior monitoring, and low levels of psychological control, indicating that a delicate balance between the parenting dimensions is crucial for the development of adolescents. The study highlights the need to develop effective school and community-based interventions to improve gender-specific paternal and maternal parenting practices with emphasis on behavior monitoring for boys and parent-child connectedness for girls, and calls for advocacy of paternal engagement in parenting, especially for girls. Family service centers, schools, communities and organizations related to child and adolescent development should provide parenting guidance to families facing challenges, helping parents build supportive environments to their children.

## Data Availability

The datasets used and/or analyzed during the current study are available from the corresponding author on reasonable request.

## References

[B1] Abdul Gafor K. Kurukkan A. (2014). Construction and validation of scale of parenting style. Guru J. Behav. Soc. Sci. 2, 315–323. Available online at: http://gjbss.org/wp-content/uploads/2015/01/GJBSS_Vol2_Issue4_Paper5_Gafor_21.pdf

[B2] Allen J. P. Chango J. Szwedo D. Schad M. Marston E. (2012). Predictors of susceptibility to peer influence regarding substance use in adolescence. Child Dev. 83, 337–350. doi: 10.1111/j.1467-8624.2011.01682.x 22188526 PMC3390749

[B3] Allen J. P. Hauser S. T. O'Connor T. G. Bell K. L. (2002). Prediction of peer-rated adult hostility from autonomy struggles in adolescent-family interactions. Dev. Psychopathol. 14, 123–137. doi: 10.1017/S0954579402001074 11893089 PMC1551977

[B4] Barber B. K. Stolz H. E. Olsen J. A. (2005). Parental support, psychological control, and behavioral control: assessing relevance across time, culture, and method. Monogr. Soc. Res. Child Dev. 70, 1–137. doi: 10.1111/j.1540-5834.2005.00365.x 16359423

[B5] Baumrind D. (1966). Effects of authoritative parental control on child behavior. Child Dev. 37, 887–907. doi: 10.2307/1126611

[B6] Baumrind D. (1967). Child care practices anteceding three patterns of preschool behavior. Genet. Soc. Gen. Psychol. Monogr. 75, 43–88. 6032134

[B7] Baumrind D. (1971). Current patterns of parental authority. Dev. Psychol. 4, 1–103. doi: 10.1037/h0030372

[B8] Blum R. W. Halcón L. Beuhring T. Pate E. Campell-Forrester S. Venema A. (2003). Adolescent health in the Caribbean: risk and protective factors. Am. J. Public Health 93, 456–460. doi: 10.2105/AJPH.93.3.456 12604495 PMC1447763

[B9] Bowling S. W. Werner-Wilson R. J. (2000). Father-daughter relationships and adolescent female sexuality. J. HIV/AIDS Prevent. Educ. Adolesc. Child. 3, 5–28. doi: 10.1300/J129v03n04_02

[B10] Bronfenbrenner U. (1986). Ecology of the family as a context for human development: research perspectives. Dev. Psychol. 22, 723–742. doi: 10.1037/0012-1649.22.6.723

[B11] CFPA (2020). Report on Reproductive Health in China. Beijing: Intellectual Property Publishing House.

[B12] Chen H. (2023). How do sibling gender structure difference affect the availability of household lending? —A new explanation for the phenomenon of “value male over female” Shandong University, China.

[B13] Cross S. E. Madson L. (1997). Models of the self: self-construals and gender. Psychol. Bull. 122, 5–37. doi: 10.1037/0033-2909.122.1.5 9204777

[B14] Deng L. Liu D. Xu J. (2018). Parental monitoring and adolescents‘ self-control: the moderating effect of fathers' self-control. Chin. J. Sp. Educ. 11, 83–91. doi: 10.3969/j.issn.1007-3728.2018.11.014

[B15] Dufur M. J. Hoffmann J. P. Erickson L. D. (2018). Single parenthood and adolescent sexual outcomes. J. Child Fam. Stud. 27, 802–815. doi: 10.1007/s10826-017-0938-7

[B16] Ephross P. H. (1980). Adolescent sexuality in a changing American Society: social and psychological perspectives. Catherine S. Chilman. Soc. Serv. Rev. 54, 143–145. doi: 10.1086/643813

[B17] Feldman S. S. Turner R. A. Araujo K. (1999). Interpersonal context as an influence on sexual timetables of youths: gender and ethnic effects. J. Res. Adolesc. 9, 25–52. doi: 10.1207/s15327795jra0901_2

[B18] Finkenauer C. Engels R. C. M. E. Baumeister R. F. (2005). Parenting behaviour and adolescent behavioural and emotional problems: the role of self-control. Int. J. Behav. Dev. 29, 58–69. doi: 10.1080/01650250444000333

[B19] Fung J. Lau A. S. (2012). Tough love or hostile domination? Psychological control and relational induction in cultural context. J. Family Psychol. 26, 966–975. doi: 10.1037/a0030457 23106102

[B20] Gilligan C. (1993). In a Different Voice sychological Theory and Women's Development. Cambridge: Harvard University Press. doi: 10.4159/9780674037618

[B21] Gillmore M. R. Chen A. C. Haas S. A. Kopak A. M. Robillard A. G. (2011). Do family and parenting factors in adolescence influence condom use in early adulthood in a multiethnic sample of young adults? J. Youth Adolesc. 40, 1503–1518. doi: 10.1007/s10964-011-9631-0 21279676 PMC5528150

[B22] Helwig C. C. To S. Wang Q. Liu C. Yang S. (2014). Judgments and reasoning about parental discipline involving induction and psychological control in China and Canada. Child Dev. 85, 1150–1167. doi: 10.1111/cdev.12183 24936611

[B23] Hoffman L. W. (1977). Changes in family roles, socialization, and sex differences. Am. Psychol. 32, 644–657. doi: 10.1037/0003-066x.32.8.644

[B24] Hosek S. Pettifor A. (2019). HIV prevention interventions for adolescents. Curr. HIV/AIDS Rep. 16, 120–128. doi: 10.1007/s11904-019-00431-y 30707399 PMC6703904

[B25] Jakobsen R. (1997). Stages of progression in noncoital sexual interactions among young adolescents: an application of the mokken scale analysis. Int. J. Behav. Dev. 21, 537–553. doi: 10.1080/016502597384776

[B26] Jin H. Yang P. Yang T. (2021). The influence of adolescents' romantic relationship on individual development: evidence from China. Int. J. Chin. Educ. 10:22125868211070036. doi: 10.1177/22125868211070036

[B27] Kapungu C. T. Holmbeck G. N. Paikoff R. L. (2006). Longitudinal association between parenting practices and early sexual risk behaviors among urban African American Adolescents: the moderating role of gender. J. Youth Adolesc. 35, 783–794. doi: 10.1007/s10964-006-9102-1

[B28] Kincaid C. Jones D. J. Sterrett E. McKee L. (2012). A review of parenting and adolescent sexual behavior: the moderating role of gender. Clin. Psychol. Rev. 32, 177–188. doi: 10.1016/j.cpr.2012.01.002 22366393 PMC3694320

[B29] Kortsmit K. Nguyen A. T. Mandel M. G. Hollier L. M. Ramer S. Rodenhizer J. Whiteman M. K. (2023). Abortion Surveillance - United States, 2021. MMWR Surveill Summ. 72, 1–29. doi: 10.15585/mmwr.ss7209a1 37992038 PMC10684357

[B30] Kushal S. A. Amin Y. M. Reza S. Hossain F. B. Shawon M. S. R. (2022). Regional and sex differences in the prevalence and correlates of early sexual initiation among adolescents aged 12-15 years in 50 countries. J. Adolesc. Health 70, 607–616. doi: 10.1016/j.jadohealth.2021.10.027 34895994

[B31] Li J. (2017). Study on the relationship between family rearing patterns*, parental attachment and psychosexual health in secondary vocational school students*. (Kunming: Yunnan Normal University).

[B32] Li X. (2013). A Study on the relationship of parenting styles, achievement motivation and academic achievement among senior high school student. (Wuhan: Center China Normal University).

[B33] Li Y. Liu F. (2004). Analysis of social attitudes towards antemarital sexual behaviors among Chinese youth. J. Nanjing College Popu. Program. Manag. 20, 23–27. doi: 10.14132/j.2095-7963.2004.01.006

[B34] Liang M. Simelane S. Fortuny Fillo G. Chalasani S. Weny K. Salazar Canelos P. . (2019). The State of adolescent sexual and reproductive health. J. Adolesc. Health 65, S3–S15. doi: 10.1016/j.jadohealth.2019.09.015 31761002

[B35] Liang X. J. Huang Z. X. Mai Z. H. B K,. T. Zhang S. S. (2008). Adolescent health risk behavior in Foshan:IV sexual behavior easily causing unintended pregnancy and sexually transmitted diseases. Chin. J. Sch. Doctor 04, 367–371. doi: 10.3969/j.issn.1001-7062.2008.04.001

[B36] Liu Y. (2020). The Effect of parental psychological control on iest anxiety of high school students: the mediating role of negative perfectionism and the moderating role of gender (Shijiazhuang: Hebei Normal University).

[B37] Liu Z. Bai J. Guo Y. Zhou N. Yu M. (2017). AIDS knowledge awareness and sexual behavior characteristics of students in the secondary vocational schools in Tianjin. Chin. J. Hum. Sex. 26, 141–143. doi: 10.3969/j.issn.1672-1993.2017.02.047

[B38] Maccoby E. E. (1990). Gender and relationships. A developmental account. Am. Psychol. 45, 513–520. doi: 10.1037/0003-066X.45.4.513 2186679

[B39] Maccoby E. E. Martin J. A. (1983). “Socialization in the context of the family: Parent-child interaction, in Handbook of Child Psychology: Vol. 4. Socialization, Personality, and Social Development, ed. M. Hetherington (New York, NY: Wiley), 1–101.

[B40] Mandara J. (2003). The typological approach in child and family psychology: a review of theory, methods, and research. Clin. Child Fam. Psychol. Rev. 6, 129–146. doi: 10.1023/A:1023734627624 12836581

[B41] Mattebo M. Tydén T. Häggström-Nordin E. Nilsson K. W. Larsson M. (2018). Pornography consumption and psychosomatic and depressive symptoms among Swedish adolescents: a longitudinal study. Upsala J. Med. Sci. 123, 237–246. doi: 10.1080/03009734.2018.1534907 30411651 PMC6327603

[B42] Mendle J. Harden K. P. Turkheimer E. Van Hulle C. A. D'Onofrio B. M. Brooks-Gunn J. . (2009). Associations between father absence and age of first sexual intercourse. Child Dev. 80, 1463–1480. doi: 10.1111/j.1467-8624.2009.01345.x 19765012 PMC2939716

[B43] Miller B. C. Norton M. C. Curtis T. Hill E. J. Schvaneveldt P. Young M. H. (1997). The timing of sexual intercourse among adolescents: family, peer, and other antecedents. Youth Soc. 29, 54–83. doi: 10.1177/0044118X97029001003

[B44] Molanos R. (2025). How parenting styles influence adolescent behavior: a closer look at developmental outcomes. J. Child Adolesc. Behav. 13:2. Available online at: https://www.omicsonline.org/open-access/how-parenting-styles-influence-adolescent-behavior-a-closer-look-at-developmental-outcomes-137039.html?view=mobile

[B45] NBSPRC (2023). China Statistical Yearbook-2023. Available online at: https://www.stats.gov.cn/sj/ndsj/ (Accessed March 2, 2025).

[B46] Ngom P. Magadi M. A. Owuor T. (2003). Parental presence and adolescent reproductive health among the Nairobi urban poor. J. Adolesc. Health 33, 369–377. doi: 10.1016/S1054-139X(03)00213-1 14596958

[B47] Nie Y. Zheng R. M. Luo X. M. Xu Y. W. (2022). Investigation and analysis of sexual development situation and knowledge, attitude and behavior about sexual and reproductive health of 15415 adolescents in China. Chin. J. Woman Child Health 33, 68–74.

[B48] Nieh H. P. Chang L. Y. Chang H. Y. Chiang T. L. Yen L. L. (2020). Pubertal timing, parenting style, and trajectories of pornography use in adolescence: peer pornography use as the mediator. J. Sex Res. 57, 29–41. doi: 10.1080/00224499.2019.1623163 31215794

[B49] Oudekerk B. A. Allen J. P. Hafen C. A. Hessel E. T. Szwedo D. E. Spilker A. (2014). Maternal and paternal psychological control as moderators of the link between peer attitudes and adolescents' risky sexual behavior. J. Early Adolesc. 34, 413–435. doi: 10.1177/0272431613494007 25328265 PMC4201509

[B50] Pirrone D. Zondervan-Zwijnenburg M. Reitz E. van den Eijnden R. Ter Bogt T. F. M. (2022). Pornography use profiles and the emergence of sexual behaviors in adolescence. Arch. Sex. Behav. 51, 1141–1156. doi: 10.1007/s10508-021-02140-3 34811657 PMC8888502

[B51] Power T. G. (2013). Parenting dimensions and styles: a brief history and recommendations for future research. Child. Obes. 9 (Suppl 1), S14–S21. doi: 10.1089/chi.2013.0034 23944920 PMC3746212

[B52] Romm K. F. Metzger A. Alvis L. M. (2020). Parental psychological control and adolescent problematic outcomes: a multidimensional approach. J. Child Family Stud. 29, 195–207. doi: 10.1007/s10826-019-01545-y

[B53] Sheeber L. B. Davis B. Leve C. Hops H. Tildesley E. (2007). Adolescents' relationships with their mothers and fathers: associations with depressive disorder and subdiagnostic symptomatology. J. Abnorm. Psychol. 116, 144–154. doi: 10.1037/0021-843X.116.1.144 17324025 PMC2249923

[B54] Shek D. T. (2008). Predictors of perceived satisfaction with parental control in Chinese adolescents: a 3-year longitudinal study. Adolesc. 43, 153–164. 18447087

[B55] Shek D. T. L. (2008). Perceived parental control and parent–child relational qualities in early adolescents In Hong Kong: parent gender, child gender and grade differences. Sex Roles 58, 666–681. doi: 10.1007/s11199-007-9371-5

[B56] Sidze E. M. Elungata‘a P. Maina B. W. Mutua M. M. (2015). Does the quality of parent-child connectedness matter for adolescents' sexual behaviors in Nairobi informal settlements? Arch. Sex. Behav. 44, 631–638. doi: 10.1007/s10508-014-0402-3 25501658

[B57] Sieverding J. A. Adler N. Witt S. Ellen J. (2005). The influence of parental monitoring on adolescent sexual initiation. Arch. Pediatr. Adolesc. Med. 159, 724–729. doi: 10.1001/archpedi.159.8.724 16061779

[B58] Sneed C. D. Amy S. Chrystene N. Morisky D. E. (2009). The influence of parental monitoring and communication on adolescents sexual behavior and intentions. Vulner. Child. Youth Stud. 4, 37–47. doi: 10.1080/17450120802460092

[B59] Song Y. (2023). Focusing on puberty development and strengthening adolescent sexual and reproductive health education. China J. Women Child Health Res. 14, 1–5. doi: 10.19757/j.cnki.issn1674-7763.2023.06.001

[B60] Stanley L. Kellermanns F. W. Zellweger T. M. (2017). Latent profile analysis: understanding family firm profiles. Family Bus. Rev. 30, 84–102. doi: 10.1177/0894486516677426

[B61] Ulferts H. (2020). Why parenting matters for children in the 21st century: an evidence-based framework for understanding parenting and its impact on child development. OECD Education Working Paper, No. 222.

[B62] van de Bongardt D. de Graaf H. Reitz E. Deković M. (2014). Parents as moderators of longitudinal associations between sexual peer norms and Dutch adolescents' sexual initiation and intention. J. Adolesc. Health 55, 388–393. doi: 10.1016/j.jadohealth.2014.02.017 24755140

[B63] Van Lissa C. J. Keizer R. Van Lier P. A. C. Meeus W. H. J. Branje S. (2019). The role of fathers‘ versus mothers' parenting in emotion-regulation development from mid-late adolescence: disentangling between-family differences from within-family effects. Dev. Psychol. 55, 377–389. doi: 10.1037/dev0000612 30570297

[B64] Wang B. Stanton B. Deveaux L. Li X. Lunn S. (2015). Dynamic relationships between parental monitoring, peer risk involvement and sexual risk behavior among bahamian mid-adolescents. Int. Perspect. Sex. Reproduct. Health 41, 89–98. doi: 10.1363/4108915 26308261 PMC4567275

[B65] Wang J. Liu A. Niu Z. (2017). The impact of father's absence on adolescent girls' early sexual behavior. Chin. J. Sch. Health 38, 1438–1440. doi: 10.16835/j.cnki.1000-9817.2017.09.052

[B66] Wang L. H. Han Y. Kong W. S. Li Z. Leng P. H. Zhuang L. R. . (2025). The relationship between romantic status and non-suicidal self-injury among vocational school students: the mediating role of difficulties in emotion regulation. Modern Prevent. Med. 52, 1235–1240. doi: 10.20043/j.cnki.MPM.202410200

[B67] Wang Q. Pomerantz E. M. Chen H. (2007). The role of parents‘ control in early adolescents' psychological functioning: a longitudinal investigation in the United States and China. Child Dev. 78, 1592–1610. doi: 10.1111/j.1467-8624.2007.01085.x 17883450

[B68] Wang S. Zhang Z. Yan L. Yang C. (2012). Survey on health-related risk behaviors among adolescents in Yanqing County, Beijing. Chin. J. Public Health 28, 113–114.

[B69] Wang T. Si L. Jiang Q. (2023). Induced abortions among Chinese adolescent girls. BMC Womens Health 23:597. doi: 10.1186/s12905-023-02754-w 37957592 PMC10644521

[B70] Wight D. Williamson L. Henderson M. (2006). Parental influences on young people's sexual behaviour: a longitudinal analysis. J. Adolesc. Health 29, 473–494. doi: 10.1016/j.adolescence.2005.08.007 16213580

[B71] Wood J. J. McLeod B. D. Sigman M. Hwang W. C. Chu B. C. (2003). Parenting and childhood anxiety: theory, empirical findings, and future directions. J. Child Psychol. Psychiatr. 44, 134–151. doi: 10.1111/1469-7610.00106 12553416

[B72] World Health Organization (2007). Helping Parents in Developing Countries Improve Adolescents' Health. Geneva.

[B73] World Health Organization (2024). Adolescent pregnancy. Available online at: https://www.who.int/news-room/fact-sheets/detail/adolescent-pregnancy (Accessed October 20, 2025).

[B74] Xiao N. Gu H. He D. Qin M. Huang J. (2022). Sexual behavior and the influential factors among unmarried adolescents aged 15-24 in Chongqing. Chin. J. AIDS STD 28, 905–909. doi: 10.13419/j.cnki.aids.2022.08.06

[B75] Xu L. (2018). Research on the knowledge, belief and behavior of reproductive health among college students in Guizhou Province from the perspective of gender (Guiyang: Guizhou University).

[B76] Xu Y. Zhang L. Hee P. (2013). “Parenting practices and shyness in Chinese children,” in Parenting across Cultures: Childrearing, Motherhood and Fatherhood in Non-Western Cultures, ed. H. Selin (Dordrecht: Springer), 13–24. doi: 10.1007/978-94-007-7503-9_2

[B77] Yaffe Y. (2023a). Maternal and Paternal Authoritarian Parenting and Adolescents' Impostor Feelings: the mediating role of parental psychological control and the moderating role of child's gender. Children (Basel) 10:10020308. doi: 10.3390/children10020308 36832436 PMC9955030

[B78] Yaffe Y. (2023b). Systematic review of the differences between mothers and fathers in parenting styles and practices. Curr. Psychol. 42, 16011–16024. doi: 10.1007/s12144-020-01014-6

[B79] Yang Y. (2025). Reciprocal relationships between parental involvement and academic performance in early adolescence: a two-year longitudinal study in China. J. Youth Adolesc. 54, 876–889. doi: 10.1007/s10964-024-02102-7 39460834 PMC11933182

[B80] Yang Y. Jiang J. (2023). Influence of family structure on adolescent deviant behavior and depression: the mediation roles of parental monitoring and school connectedness. Public Health 217, 1–6. doi: 10.1016/j.puhe.2023.01.013 36812808

[B81] Yimer B. Ashebir W. (2019). Parenting perspective on the psychosocial correlates of adolescent sexual and reproductive health behavior among high school adolescents in Ethiopia. Reprod. Health 16:66. doi: 10.1186/s12978-019-0734-5 31113436 PMC6528244

[B82] Yin H. (2023). Study on the influence of junior high school students' perception of family upbringing education on their sexual mental health and educational countermeasures (Kunming: Yunnan Normal University).

[B83] Yin K. Peng J. Zhang J. (2020). The application of latent profile analysis in organizational behavior research. Adv. Psychol. Sci. 28, 1056–1070. doi: 10.3724/SP.J.1042.2020.01056

[B84] Yu C. Lou C. Lian Q. Tu X. Zhang J. Zuo X. (2021). The pattern of romantic and sexual related experiences among Chinese young adolescents: an exploration with multi-group latent class analysis. Reprod. Health 18:184. doi: 10.1186/s12978-021-01235-3 34544440 PMC8454020

[B85] Zhang L. (2019). The effects of parental control on adolescents' aggressive behavior: a moderated mediating model (Wuhu: Anhui Normal University).

[B86] Zhang P. (2012). The relationship of parental control and adolescent problem behavior. Jinan: Shandong Normal University.

[B87] Zhang X. He Y. Xu S. Pan X. Yang X. (2013). A survey on sexual and reproductive health knowledge, attitudes and behaviors among secondary vocational school students: taking students from three secondary vocational schools in Harbin as an example. Heihe J. 4, 186–188. doi: 10.14054/j.cnki.cn23-1120/c.2013.04.075

[B88] Zhao H. Liu H. Wang L. Yang X. Wang S. Han M. Li J. (2020). Epidemiological characteristics of newly-reported hiv cases among youth aged 15-24 years - China, 2010-2019. China CDC Wkly 2, 913–916. doi: 10.46234/ccdcw2020.249 34594799 PMC8422364

[B89] Zhao R. Zhang L. Fu X. X. Su C. Y. Zhang Y. P. (2019). Sexual and reproductive health related knowledge, attitude and behavior among senior high school and college students in 11 provinces and municipalities of China. Chin. J. Public Health 35, 1330–1338. doi: 10.11847/zgggws1124531

[B90] Zheng Y. Fang Y. Jin Y. Zuo X. Lian Q. Lou C. Yu C. Tu X. Li L. Hong P. (2022). Parenting practice profiling and its associated factors among secondary vocational school students in China. Int. J. Environ. Res. Public Health 19:19127497. doi: 10.3390/ijerph19127497 35742746 PMC9223670

[B91] Zhou Q. Jin C.-Y. Wang H.-J. (2021). Sexual and Reproductive Health in China. Oxford: Oxford University Press. doi: 10.1093/acrefore/9780190632366.013.224

